# Activation of Anti-Tumor Immune Response and Reduction of Regulatory T Cells with *Mycobacterium indicus pranii* (MIP) Therapy in Tumor Bearing Mice

**DOI:** 10.1371/journal.pone.0025424

**Published:** 2011-09-30

**Authors:** Faiz Ahmad, Jiju Mani, Pawan Kumar, Seenu Haridas, Pramod Upadhyay, Sangeeta Bhaskar

**Affiliations:** Product Development Cell-I, National Institute of Immunology, New Delhi, India; Fundació Institut Germans Trias i Pujol- Universitat Autònoma de Barcelona CibeRES, Spain

## Abstract

**Background:**

Role of immune system in protecting the host from cancer is well established. Growing cancer however subverts immune response towards Th2 type and escape from antitumor mechanism of the host. Activation of both innate and Th1 type response is crucial for host antitumor activity. In our previous study it was found, that *Mycobacterium indicus pranii* (MIP) also known as *M. w* induces Th1 type response and activates macrophages in animal model of tuberculosis. Hence, we studied the immunotherapeutic potential of MIP in mouse tumor model and the underlying mechanisms for its antitumor activity.

**Methodology and Principal Findings:**

Tumors were implanted by injecting B16F10 melanoma cells subcutaneously into C57BL/6 mice. Using the optimized dose and treatment regimes, anti-tumor efficacy of heat killed MIP was evaluated. In MIP treated group, tumor appeared in only 50–60% of mice, tumor growth was delayed and tumor volume was less as compared to control. MIP mediated immune activation was analysed in the tumor microenvironment, tumor draining lymph node and spleen. Induction of Th1 response and higher infiltration of immune cells in the tumor microenvironment was observed in MIP treated mice. A large fraction of these immune cells were in activated state as confirmed by phenotypic and functional analysis. Interestingly, percentage of Treg cells in the tumor milieu of treated mice was less. We also evaluated efficacy of MIP along with chemotherapy and found a better response as compared to chemotherapy alone.

**Conclusion:**

MIP therapy is effective in protecting mice from tumor. It activates the immune cells, increases their infiltration in tumor, and abrogates tumor mediated immune suppression.

## Introduction

Immune system plays a crucial role in protecting the host against cancer. There is strong evidence for the existence of an effective cancer immunosurveillance process in human and mice. In tumor bearing host however, immune system is often not able to mount an effective response, primarily because of negative regulatory mechanisms employed by growing cancer [1]. Th1 branch of the immune system, which employs T cells, NK cells and macrophages, play major role in combating cancer but growing cancers actively suppress immune response and disregulates the activity of these effector cells [2], [3].

Since long, bacteria and bacterial products have been tried for the treatment of cancer. Starting from the practical observation of tumor regression in individuals with concomitant bacterial infection and systematic efforts of William Coley in this direction, the field has developed into some standard clinical practices, such as the use of BCG for the treatment of superficial bladder cancer for over 25 years [4]. BCG-cell wall components has been administered to patients for postoperative treatment of cancer, producing good prognosis [5], [6]. *Mycobacterium vaccae* was also studied in few clinical trials [7]. Indeed, it has been observed that different mycobacterial species differ widely in their antitumor potential and there is need of sincere efforts to look for potent candidate species. A related organism *Mycobacterium indicus pranii* (MIP) is non-pathogenic, soil derived, rapidly growing, atypical mycobacterium [8]. Polyphasic taxonomic analysis has established it as a distinct species [9]. There are some key differences between BCG and MIP. BCG is attenuated form of pathogenic organism *M. bovis*, survives in host cells for months [10] and has been shown to cause toxicity in some patients while MIP is cleared from the host body in few weeks and is non-pathogenic [11].

Couple of small clinical studies, where MIP was given to lung cancer and bladder cancer patients, indicated its beneficial effect in the management of the disease [12], [13]. In animal model of tuberculosis we had found that MIP induces Th1 type response as indicated by the activation of both CD4+ and CD8+ T cells and also macrophages which control growth and multiplication of *M.tb* [11]. As induction of Th1 type of immune response is crucial to overcome the immuno-suppressive tumor microenvironment, we sought to analyse the immunotherapeutic potential of MIP in mouse model of tumor. It was observed that tumor growth was delayed and volume of tumors were significantly less in the MIP treated group as compared to control. Tumor appeared in only 50–60% of the mice in MIP treated group and in these mice tumors were infiltrated with higher number of CD4+ and CD8+T cells, NK and NKT cells, macrophages and dendritic cells. The immune cells were in functionally active state in the MIP treated group, as there was higher induction of proinflammatory cytokines and increased cytotoxicity towards target tumor cells as compared to control. Further, significantly less percentage of regulatory T cells were found in the tumor mass of MIP treated mice as compared to control tumor bearing mice.

## Materials and Methods

### Animal

Inbred C57BL/6 mice at 6–8 weeks of age were obtained from the animal facility of the National Institute of Immunology, New Delhi, India, where animals are bred and housed in agreement with the guidelines of the Institute's Animal Ethics Committee. All animal experiments were performed in accordance with Animal Ethics Committee's guidelines (Approval ID of the project - IAEC#/205/08).

### Cell lines

B16F10 melanoma cell line (obtained from American Type Culture Collection, ATCC number: CRL-6475) was cultured in DMEM medium. YAC-1 lymphoma cells (obtained from National Centre for Cell Science, Pune, India), were cultured in RPMI 1640 medium. Culture media was supplemented with 10% FBS and 1% antibiotic-antimycotic solution, and cells were grown in 37°C incubator with 5% CO_2_/95% humidified air. These cells were free of mycoplasma contamination.

### Inactivation of *Mycobacterium indicus pranii* (MIP)

MIP, previously known as *M. w* was maintained on Lowenstein-Jensen medium (LJ) slants (BD Difco) and kept at −80°C. It was cultured in Middlebrook 7H9 medium (BD Difco) with 0.2% glycerol, 0.05% Tween 80 and 10% albumin-dextrose-catalase enrichment (BD Difco) as a shake flask culture. Bacteria were harvested in the log growth phase by centrifugation at 840×g for 15 min, washed twice with PBS, and suspended in saline at the desired concentration. These were then inactivated by autoclaving for 20 min at a pressure of 15 lb/in^2^. These inactivated bacteria were used for immunisation & treatment in the tumor bearing mice.

### Tumor implantation

Initially different numbers of B16F10 cells were subcutaneously injected in the right flank of syngeneic C57BL/6 mice to determine an appropriate tumor burden. For all subsequent experiments, 30,000 B16F10 cells were injected, which resulted in appearance of tumor in about 2 week time in all the animals. For survival studies, less number of (10,000) B16F10 cells were injected so as to observe the mice for longer time period.

### Standardisation of dose-regimen of MIP

Initial experiments were done to find out the optimum dose and treatment regimen of MIP which reduced the tumor growth reproducibly. In these experiments, MIP, suspended in PBS, was given by s.c route near the site of tumor cell injection at 7 day interval and once the tumor started appearing it was given peritumorally. Based on these studies, an optimum dose of 5×10^6^ bacilli/100 µl PBS per animal was selected. This optimum dose was then further evaluated in two treatment regimens viz therapeutic and prophylactic+therapeutic. In therapeutic treatment, MIP was given at 7 day interval starting from one day after tumor cell implantation. In prophylactic+therapeutic treatment, one injection of MIP was given before implantation of melanoma cells (prophylactic treatment) and then it was given at weekly interval after implantation of melanoma cells (therapeutic treatment). Progression of tumor was determined by measuring tumor volume and protective efficacy of MIP was compared in different groups.

### Immunological studies

Mice were euthanized by CO_2_ asphyxiation. Single cell suspension was prepared from spleen and tumor draining lymphnodes by mincing the tissue through 70 µm mesh. Erythrocytes were lysed by using the Gey's solution. These cells were restimulated with MIP antigen (sonicate supernatant of MIP) or UV-irradiated B16F10 cells for 48 hrs. Culture supernatant was analysed for different cytokines by ELISA. Cells were also studied for the expression of specific cell surface markers by FACS.

### Preparation of tumor infiltrating mononuclear cells

Subcutaneously implanted tumors when reached 400–500 mm^3^ in size, were removed from mice and single cell suspensions were prepared by enzymatic digestion. Resected tumors were weighed, minced into small (1–2 mm^3^) pieces with a scalpel, and incubated in digestion mixture [5% FBS in RPMI 1640, 0.5 mg/ml collagenase A (Roche Diagnostic), 0.2 mg/ml hyaluronidase, type V (Sigma-Aldrich), and 0.02 mg/ml DNase I, (Sigma-Aldrich)] for 45 minutes at 37°C. The suspension was filtered using 40 µm cell strainer (BD) and centrifuged at 280 g for 10 minutes. The pellet was washed and resuspended in incomplete RPMI. Cell suspension was layered over Ficoll-paque (GE Healthcare) and centrifuged at 550 g for 30 minutes at 22°C. The buffy coat at inter-phase was collected, cells were washed, and finally suspended in PBS for further studies.

### Flow cytometry

Single cell suspension of mononuclear cells obtained from tumor mass, draining lymph node and spleen were stained with Abs against various cell surface markers using standard staining methods [14]. The following panel of commercially available and directly fluorochrome conjugated anti-mouse monoclonal antibodies were used in the study. FITC anti-mouse CD3, PE anti-mouse CD4, PE anti-mouse CD8, FITC anti-mouse CD11c, FITC anti-mouse CD14, PE anti-mouse CD40, PerCP anti-mouse CD69, PE anti-mouse CD80, PE anti-mouse CD86, PE anti-mouse NK1.1, and PE anti-mouse MHCII were purchased from eBioscience or BD Biosciences. Samples were run on a BD LSR (BD Bioscience) flow cytometer and data was analysed using WinMDI 2.9 software.

### Intracellular FoxP3 staining

Single cell suspensions prepared as described above, were used for intracellular FoxP3 staining. Staining was performed using e-Biosciences intracellular FoxP3 staining kit (cat.71-5775), following the instruction given by manufacturer. Briefly, cells were first surface stained with PE anti-mouse CD4, washed in cold PBS and suspended in freshly prepared fixation and permeabilization solution for 2 hour at 4°C in dark. Cells were thereafter washed with permeabilization buffer and incubated with FITC-anti mouse FoxP3 for 1 hr. After washing with PBS, cells were analyzed in flow cytometer.

### NK cell cytotoxicity

NK cell cytotoxicity was evaluated using YAC-1 cells as target. These cells were stained with CFSE at 2.0 µmol/ml concentration for 5 minutes. Splenocytes collected from different groups of mice were co-cultured with CFSE labelled YAC-1 cells at a ratio of 40∶1 for 6 hours at 37°C/5% CO_2_. Target cell killing was determined by flowcytometry on the basis of PI uptake. Percent cytotoxicity = 100×[No. of CFSE & PI double positive cells]÷[(No. of CFSE positive cells)+(No. of CFSE & PI double positive cells)].

### CTL activity

Cytotoxic T lymphocytes (CTLs) were derived from splenocytes by culturing them with UV irradiated B16F10 cells at ratio of 10∶1 for 5 days. The culture was supplemented with recombinant murine IL-2 (20 units/ml). After 5 days, CTLs were harvested and cocultured with CFSE labelled B16F10 cells at effector: target ratio of 40∶1 for 6 hours. Target cell killing was determined by flowcytometry on the basis of PI uptake. Percent cytotoxicity was calculated as above.

### MIP as an adjunct to chemotherapy

Drug Cyclophosphamide (CTX) was chosen for this study. CTX was given by single i.p injection 7 days after tumor cell implantation at a dose of 200 mg/kg body weight. Mice were treated with CTX alone or CTX along with weekly injection of MIP and groups were compared for progress in tumor, tumor volume and survival of animals.

### Statistical analysis

Statistical analyses were performed by a two-tailed student t-test with GraphPad InStat software (GraphPad Software Inc.). Data are represented as the mean ± SEM or mean ± SD as indicated in respective figure legends.

## Results

### Optimisation of dose-regimen of MIP

**Figure 1:**
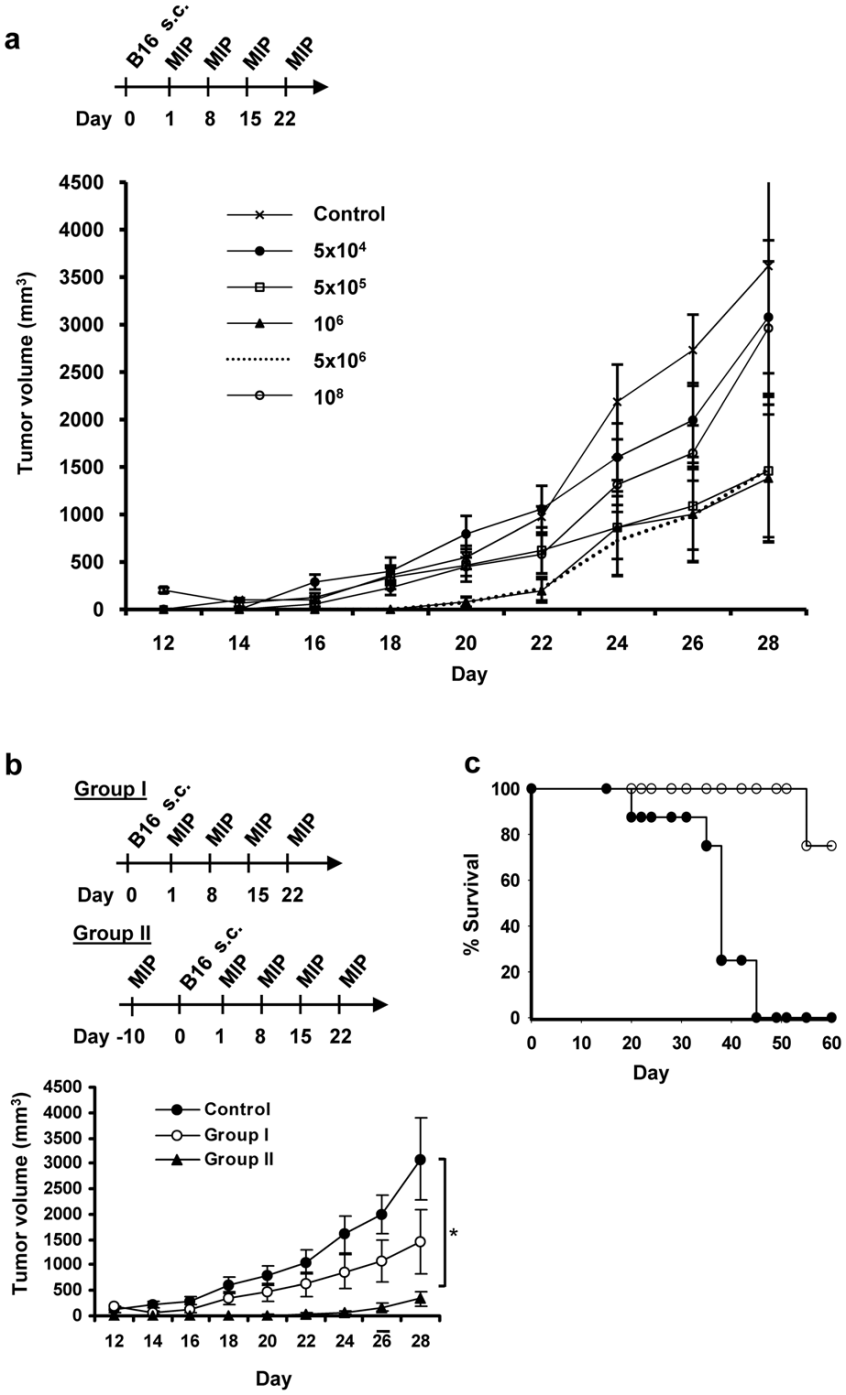
Standardisation of dose and treatment regimen of MIP. **a**) 30,000 B16F10 melanoma cells were injected in the right flank of C57BL/6 mice on day 0. MIP dose in the range of 5.0×10^4^ to 1.0×10^8^ bacilli per 100 µl of PBS was given peritumorally at 7 day interval starting from day one. Tumor growth was measured every 2 days. Control tumor bearing mice were injected saline. Dose of 5.0×10^6^ bacilli gave optimum protection. Data are mean ± SE values obtained from 8 mice/group. **b**) MIP at a dose of 5.0×10^6^ bacilli per 100 µl PBS was further evaluated using two different treatment regimens viz. therapeutic (Group I) and prophylactic+therapeutic (Group II). Treatment schedule is shown in the figure. In comparison to therapeutic regimen, prophylactic+therapeutic regimen was found to be more effective. This treatment regimen was therefore used for all the subsequent studies. **c**) For survival studies less number (10,000) of B16F10 melanoma cells were injected subcutaneously in the right flank and MIP treatment (as for Group II) was followed. Percent survival for observation period of 60 days is shown for control and MIP treated mice. (•) Control group; (○) MIP treated group. Tumor volume = 0.5×large diameter×small diameter^2^. *p<0.05.

Initial experiments were done to optimise the dose and regimen of MIP treatment. B16F10 melanoma cells were injected subcutaneously on the right flank of mice. Heat killed MIP suspended in PBS was used for the treatment. MIP concentration in the range of 5.0×10^4^ to 1.0×10^8^ per 100 µl was given peritumorally at 7 day interval, starting from day one. It was observed that lower dose of MIP (5×10^4^) and higher dose (1.0×10^8^) was less effective while the dose in the range of 0.1 million to 10 million bacilli per injection gave good antitumor response (Fig. 1a). Therefore, dose of 5.0×10^6^ bacilli/100 µl PBS per animal was chosen for subsequent studies. This dose was then further evaluated in two treatment regimens viz therapeutic (group I), and prophylactic+therapeutic (group II). In prophylactic+therapeutic regimen, one injection of MIP was given at ten day (−10) before tumor implantation, tumor was implanted on day 0, and weekly treatment followed from day 1 onwards. In therapeutic regimen, however, treatment started only after tumor cell implantation. Both regimens, reduced the tumor growth as compared to control group but, tumor growth was substantially delayed and volume of tumors were significantly less in the Group II (Fig. 1b). In control mice, tumor started appearing about 2 week after tumor cell implantation, while in the mice vaccinated with MIP, it started appearing only after 3 week of tumor cell injection and tumor appeared in only 50–60% of mice. Therefore, in all subsequent experiments a dose of 5.0×10^6^ bacilli per 100 µl was used, and prophylactic+therapeutic treatment regimen was followed.

After this we compared the survival of MIP treated and control mice. For survival studies less number (10,000) of B16F10 cells were injected and MIP treatment followed as for Group II. In MIP treated mice, there was delayed tumor appearance and growth of tumor was also slow. Consequently survival of MIP treated tumor bearing mice was increased. While, in the control group, all the mice died during the experimental observation period, only 25% of the mice in the vaccinated group died during this period (Fig. 1c).

### MIP induced Th1 immune response in tumor bearing mice

**Figure 2:**
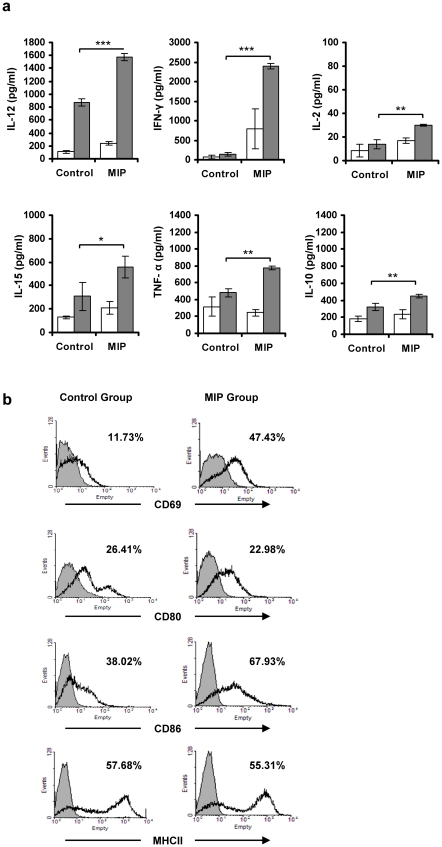
Evaluation of MIP induced modulation of immune response. Splenocytes from control and MIP treated tumor bearing mice were prepared after 3 weeks of tumor implantation and restimulated *in vitro* with MIP antigen for 48 hrs. **a**) Culture supernatants was analyzed for IL-12, IFN-γ, IL-2, IL-15, TNF-α and IL-10 by ELISA. Each bar represents the mean ± SD of the two independent experiments (n = 4). (□) unstimulated; (▪) restimulated with MIP antigen; *p<0.05; **p<0.01; ***p<0.0001. **b**) Activation of splenocytes after *in vitro* restimulation with MIP antigen. Splenocytes were isolated, restimulated *in vitro* with MIP antigen for 48 hrs and were stained with respective Abs using standard staining method. Cells were analyzed by flow cytometry. In comparison to control group, percentage of cells expressing CD69 and CD86 was higher in the MIP treated group; (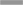) unlabelled cells; (––) labelled cells.

Th1 immune response plays a decisive role in protection from tumor. Growing tumors, however, actively suppress the immune response. We therefore evaluated the immune modulation induced by MIP in tumor bearing mice. Splenocytes isolated from control and MIP treated, tumor bearing mice were restimulated *in vitro* with soluble antigens of MIP, and culture supernatant was analysed for different proinflammatory and suppressive cytokines by ELISA. Significantly high amounts of proinflammatory cytokines, IL-12, IFN-γ and TNF-α were found in the culture supernatant of *in vitro* restimulated splenocytes from MIP treated mice as compared to control mice (Fig. 2a). IL-2 and IL-15 were found in moderate amount in both groups. Small amount of IL-10 was also induced on restimulation with MIP in both groups.

We also evaluated the phenotype of these splenocytes, restimulated *in vitro* with MIP antigen. As shown in figure 2b, there was significantly higher expression of CD86 and CD69, indicating higher activation status of antigen presenting cells and lymphocytes in MIP treated group as compared to control group. Expression of CD80 and MHCII was comparable in both groups.

### Tumors in MIP treated mice infiltrated with higher number of lymphocytes, macrophages, and dendritic cells

**Figure 3:**
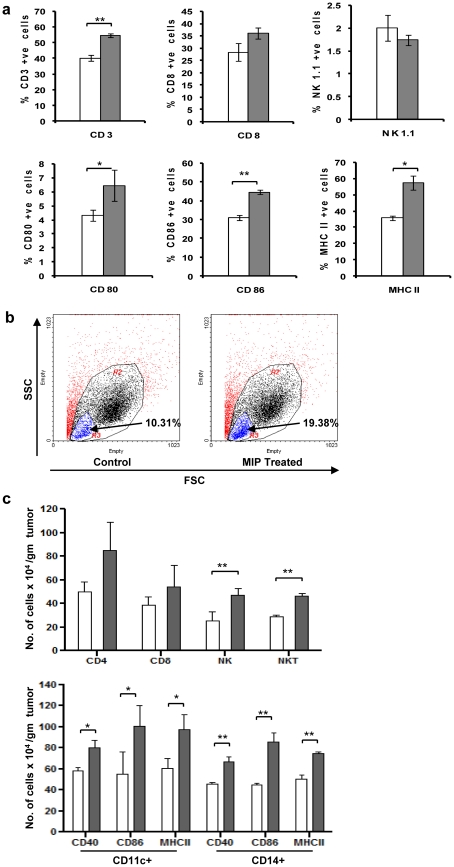
Phenotypic analysis of immune cells in tumor draining lymph node (TDLN) and Tumor. **a**) Inguinal lymph nodes were removed from tumor bearing mice two weeks after tumor cell implantation. Single cell suspension were then analysed for expression of CD3, CD8, NK1.1, CD80, CD86, and MHCII by flow cytometry. Each bar represents the mean ± SD of the two independent experiments (n = 3). **b**) Single cell suspension obtained from buffy layer of ficoll gradient, of tumor cell suspension was analyzed for immune cell infiltration. Higher infiltration of immune cells was observed in the MIP treated mice. **c**) Immune cell subtypes infiltrating the tumor. Number of specific cells (N) per gram of tumor was calculated by formula: N = NSxNT/NAxW, where NS is the number of specific immune cells in the total acquired cells on flow cytometer; NA is the total no. of acquired cells; NT is the total number of cells in the buffy coat on ficoll gradient; W is the tumor weight in gram. Tumors in MIP treated mice were found to be infiltrated with higher number of lymphocytes, activated macrophages and dendritic cells; experiment was repeated twice; each group having 6 animals. Data shown is mean of two independent experiments (mean ± SD). *p<0.05; **p<0.01; ^•^ *p* = 0.05.

For understanding the mechanism of MIP mediated tumor protection, distribution of immune cells was analysed in the tumor draining lymphnodes and tumor microenvironment of control and MIP treated, tumor bearing mice. As shown in the figure 3a, there was increase in the percentage of CD3+ and CD8+ T cells in the tumor draining lymph node of MIP treated mice. When we checked the activation status of immune cells, it was observed that expression of CD80, CD86 and MHC II was significantly high in the MIP treated group. Frequency of NK cells was comparable in both groups.

Immune response in the tumor microenvironment is most crucial factor to decide the prognosis of the disease. In general higher infiltration of immune cells correlates with better prognosis, so we analysed the tumor microenvironment for the same. About four week after tumor cell implantation, mononuclear cells from tumor mass were isolated and analysed by FACS. Tumors in MIP treated mice were found to be infiltrated with higher number of total immune cells (Fig. 3b). Since the tumor volume/weight was significantly different in the two groups, we analysed it, as the number of specific immune cells per gram of tumor. It was observed that in MIP treated group, higher number of CD4+, and CD8+ T cells were infiltrating the tumor (Fig. 3c). Similarly, the number of NK and NKT cells infiltrating the tumor of treated mice were also higher as compared to control mice.

Dendritic cells are potent antigen presenting cells and provide necessary signal for activation and proliferation of lymphocytes. Hence, we analysed the tumor infiltrating dendritic cells, which were found in higher number in MIP treated group (Fig. 3c). Most of these cells were in activated state, as evidenced by expression of CD40, CD86 and MHCII. Macrophages, besides having antigen presenting function, also show direct cytotoxicity against tumor cells. In comparison to control group, we observed higher infiltration of macrophages in the treated group and most of these cells were expressing CD40, confirming M1 phenotype of these cells, which is associated with better prognosis (Fig. 3c). Most of these cells were also expressing CD86 and MHCII, providing evidence of their activated state as compared to control group.

### MIP treated mice had less number of regulatory T cells in tumor and tumor draining lymph node

**Figure 4:**
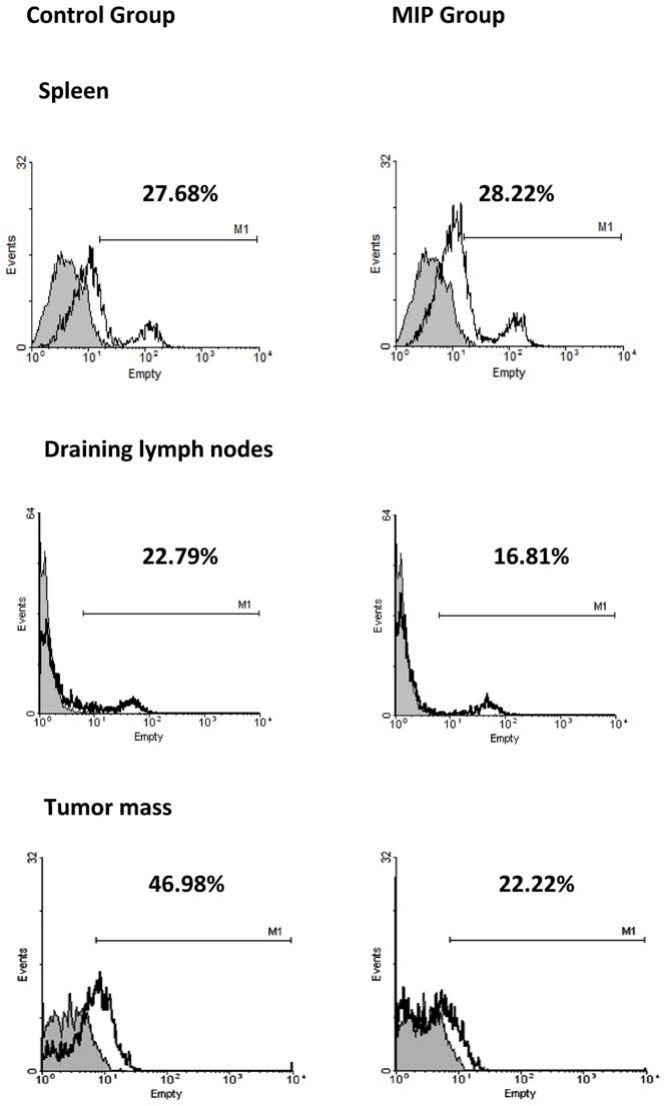
Treg cells in spleen, tumor draining lymph node and tumor mass. Single cell suspension from spleen, tumor draining lymph nodes, and tumor of control and MIP treated tumor bearing mice were analyzed for the expression of CD4 and FoxP3 by flow cytometry. In tumor microenvironment and tumor draining lymph node, the percentage of Treg cells were found to be lower in the MIP treated group as compared to control. In spleen comparable level of these cells were found in both, treated and untreated groups. (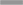) CD4+ cells; (––) CD4+FoxP3+ cells.

Regulatory T cells (Tregs) promote tumor growth by down-regulating the anti-tumor immune response. Multiple mechanisms for Treg mediated immune suppression have been established. We studied the Treg cells in spleen, tumor draining lymph node and tumor mass when the tumor reached 400–500 mm^3^ in size. In spleen we found comparable level of these cells in both treated as well as untreated groups (Figure 4). However, in the tumor draining lymphnodes and tumor microenvironment, Treg cell percentage was lower in the MIP treated group. Treg cell expansion in the tumor of control mice was substantial. While percentage of these cells was nearing 50% of the total CD4+ cells in the tumor infiltrating lymphocytes of control mice, it was just half in the MIP treated group.

### MIP treatment induced substantial antitumor immune response

**Figure 5:**
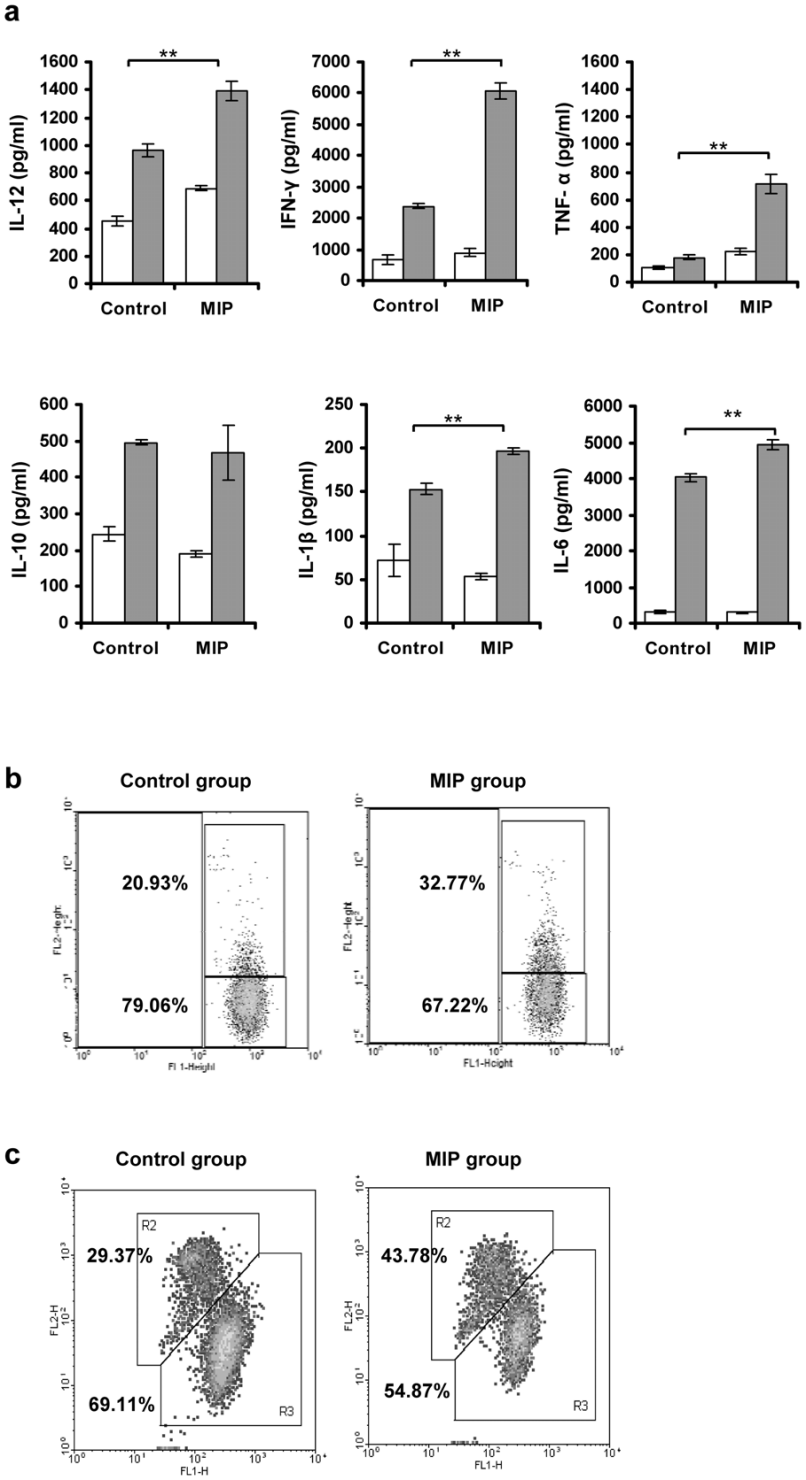
Tumor cell specific immune response. **a**) Splenocytes from control and MIP treated tumor bearing mice at 3 weeks after tumor implantation were in-vitro restimulated with UV-irradiated B16F10 melanoma cells for 48 hrs. Culture supernatants were then analyzed for the levels of IL-12, IFN-γ, TNF-α, IL-10, IL-1β and IL-6 by ELISA. Each bar represents the mean ± SD of the two independent experiments (n = 4); (□) unstimulated; (▪) restimulated; **p<0.01. **b**) NK cell cytotoxicity. To check NK cell cytotoxicity, CFSE labeled Yac-1 target cells were co-cultured with splenocytes from control and MIP treated tumor bearing mice for 6 hrs. Target cell killing was analysed on the basis of PI uptake by flow cytometry. Cytotoxicity was found to be higher in the MIP treated group as compared to control. **c**) CTL response against B16 melanoma cells. CTLs were derived from splenocytes of control and MIP treated tumor bearing mice by culturing with UV-irradiated B16F10 cells and rIL-2 for 5 days. Cytotoxicity was determined by co-culturing CTLs with CFSE-labeled B16F10 target cells. % Cytotoxicity = 100×[No. of CFSE & PI double positive cells]÷[(No. of CFSE positive cells)+(No. of CFSE & PI double positive cells)].

After studying the infiltration and phenotypic activation markers of various immune cells, we studied the functional status of these cells in the control or MIP treated tumor bearing mice, three week after implantation of melanoma cells. First we evaluated the tumor cell specific recall response. Mononuclear cells isolated from the spleen of both groups were restimulated *in vitro* with UV-irradiated B16 cells, which provided whole range of antigens present in B16 cells and supernatants were analysed for the cytokines. Proinflammatory cytokines IFN-γ, TNF-α, IL-12 and IL-6 were found to be significantly high in the B16 stimulated lymphocytes from MIP treated group as compared to control group (Fig. 5a). IL-1β and IL-10 were induced in response to stimulation with B16 cells in both the groups but the amounts of cytokines secreted were moderate. These findings confirmed that immune cells from MIP treated group were functionally active and secreted proinflammatory cytokines crucial for anti-tumor response.

We then analysed the cytolytic activity of NK cells and T cells. These cells have potent cytolytic activity against tumor cells and are crucial for protecting the host from neoplastic diseases. NK cell cytotoxicity towards YAC-1 cells, as determined by flowcytometry, was found to be higher in the MIP treated group as compared to control (Fig. 5b). Similar observation was made when *in vitro* generated CTL were co-cultured with B16F10 target cells. CTL activity was found to be higher in the MIP treated group as compared to control untreated group (Fig. 5c). These results confirm that MIP treatment in the tumor bearing mice results in functional activation of T cells and NK cells which along with other factors results in regression of tumor.

### Efficacy of MIP as an adjunct to chemotherapy

**Figure 6:**
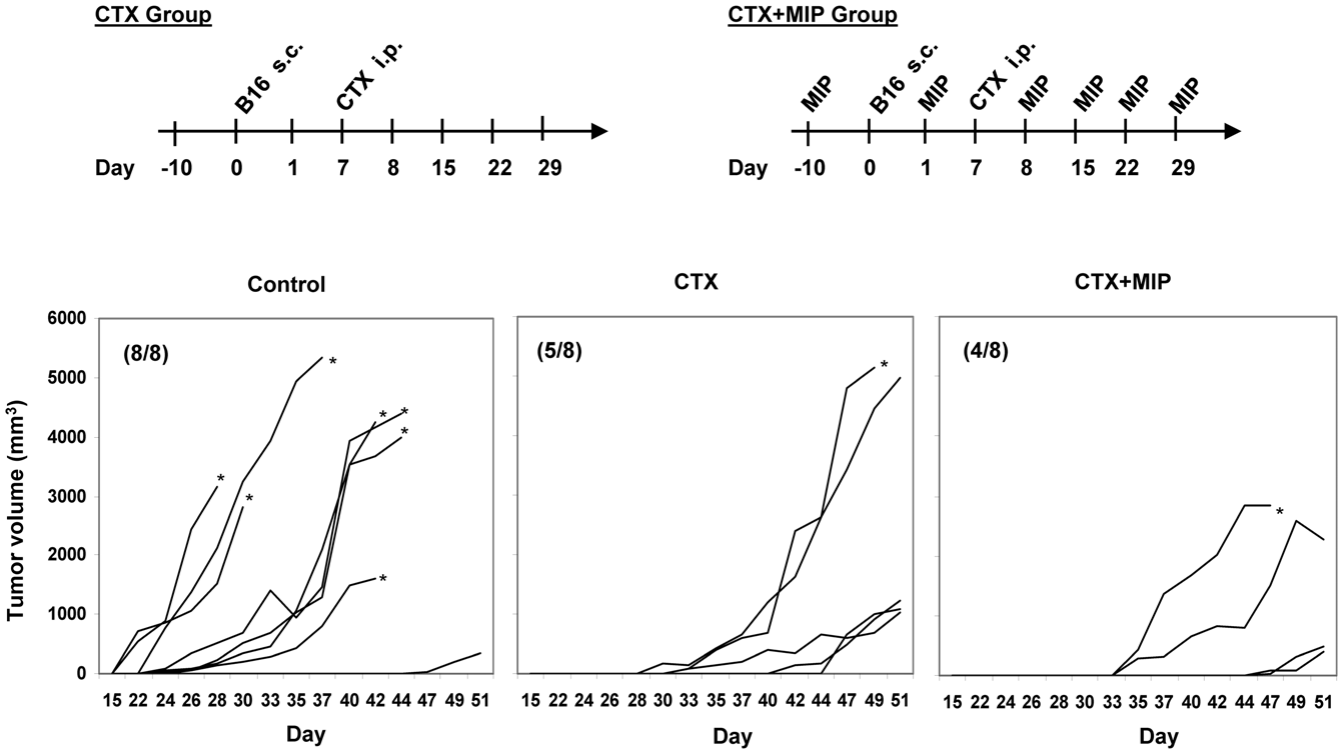
Antitumor activity of MIP given with Cyclophosphamide. Mice were treated with CTX alone or CTX along with MIP (CTX+MIP). CTX was given intraperitoneally on day 7th after tumor cell implantation at a dose of 200 mg/kg in both groups. In CTX+MIP group, MIP injection was given at 1 week interval as shown in figure. Each line in graph represents tumor growth curve of individual mice. MIP given along with CTX therapy showed higher protection as compared to CTX alone. 4 out of 8 mice were free of tumor and tumor volume was also less in this group. * symbolize the death of mice on the corresponding day. Number in parenthesis indicates number of mice showing tumor out of 8 mice.

To explore the use of MIP in real life situation, we evaluated its efficacy as an adjunct to chemotherapy. Cyclophosphamide, an anti-cancer drug used for the treatment of lymphomas, leukemias, and solid tumors was chosen for this study. Mice were treated with cyclophasphamide alone (CTX group) or cyclophasphamide along with MIP (CTX+MIP group) and were observed for growth of tumor (Fig. 6). In the control group, tumor began to appear at 2–3 week after tumor cell implantation. In CTX group, there was delay in tumor growth and it started appearing only at 4–5 week after tumor cell implantation. But in the group where MIP was given along with CTX, tumor growth was significantly less and 4 out of 8 mice remained free from tumor for the experimental observation period and 2 mice had very small tumor growth. In control group all the mice developed tumor and died of the tumor burden whereas, only one mice in the CTX+MIP group died during this time.

## Discussion

Although progress has been made in the field of tumor immunology in the past decade but the vaccines were mostly evaluated based on surrogate end points like histologic evidence of tumor necrosis or lymphocyte infiltration rather than objective tumor regression [15], [16]. In our study we have objectively studied the tumor growth kinetics along with other supporting immunological parameters.

Results of this study provide evidence of immunotherapeutic potential of MIP in mouse model of tumor. We chose mouse syngenic B16F10 melanoma cells for this study as this is very aggressive tumor and poorly immunogenic. MIP was compared for immunotherapeutic efficacy using two different treatment regimens. In therapeutic regimen, MIP was given after implantation of melanoma cells. In prophylactic+therapeutic regimen, one injection was also given before tumor implantation and rest were given as for therapeutic regimen. Decrease in tumor growth was observed in both groups as compared to control, but in the later group, growth of tumor was delayed and volume of tumor was significantly less. There was no tumor growth in about 40–50% of mice which resulted in the prolonged survival of the treated mice as compared to control.

Since 1882, when Coley used a bacterial preparation (Coley toxins) to treat sarcomas, there has been evidence that bacterial products can beneficially affect the course of several cancers. BCG has been used extensively with some definite but limited anti-tumor efficacy [17], [18], [19]. Intravesical administration of live BCG is successful for cancer of urinary bladder, but is associated with significant toxicity and is ineffective in 30–40% of cases. Hence, other mycobacterial strains have also been evaluated for their anticancer efficacy. It has been reported that closely related mycobacterial strains demonstrate contrasting levels of efficacy as antitumor vaccine as they are processed differently inside the antigen presenting cells [10]. MIP and BCG have significant differences viz. growth rate of BCG is slow, while MIP is fast grower [20]. There are structural differences in the cell wall of fast and slow-growing mycobacteria [21], [22], which might affect their intracellular processing and thus the immune response induced.

It is generally accepted that the generation of an effective anti-tumor and anti-infective immune response requires antigen presenting cells to prime cytotoxic T cells and CD4+T cells. It is reported that established tumors can be eradicated by CD8+T cell adoptive immunotherapy [23]; and extensive T cell infiltrates are commonly seen in tumors undergoing immunologic rejection [15].

We observed higher frequency as well as higher expression of phenotypic activation markers on macrophages and T cells of MIP treated mice which also reflected in their enhanced functional activity. In addition to induction of IFN-γ, TNF-α and IL-12 secretion, higher NK cell and CD8+ T cell cytotoxic activity was observed. NK cells have been shown to have important role in anti-tumor immune response. It has been shown that patients with high level of NK cell infiltration had better prognosis than those with low level of NK cells in colon and lung carcinomas [24], [25].

An interesting finding of this study was that along with activation of antigen presenting cells, NK cells and T cells, peritumoral injection of MIP also resulted in significantly less number of tumor infiltrating regulatory T cells. Proportion of regulatory T cells in the spleen was not significantly different in the MIP treated and control group and was about 20% of the total CD4+ cells. It is reported that expansion of Treg cells inside the tumor is substantial and occur to lesser extent in the spleen and lymphnodes and that too in the late stages of tumor growth [26]. Treg cells inhibit the development and effector functions of tumor-specific T cells. They accumulate in the tumor microenvironment and suppress cytotoxic T cell responses against the tumors [27], [28]. Elimination of these suppressor T cells from tumor uncovers natural antitumor response. [28], [29]. Strategy that combines modulation of suppressive factors along with the activation of pro-inflammatory Th1 type of response within the tumor microenvironment could be a potentially effective approach which could be combined with chemotherapy to combat cancer successfully.

Hence, we explored the complementary approach of combination of immunotherapy and chemotherapy to improve the anticancer efficacy of the both. We observed synergistic activity of CTX and MIP treatment. It is suggested that the mechanism of this form of immunotherapy is, at least in part due to immune modulation i.e. selective enhancement of Th1 activity. Other factor could be the TNF-α secretion in the tumor microenvironment, by MIP immunisation, which is reported to increase the permeability of tumor vessels for passage of cytotoxic drugs [30].

We also observed that MIP injection even if given at the dorsally opposite side of tumor, reduced tumor growth as compared to control. A valuable property of MIP immunotherapy is its virtual lack of adverse side effects as this has been administered to thousands of patients in clinical trials of tuberculosis, and leprosy with no reported problems [31], [32]. Another great advantage is the low cost of production.

In summary, our findings establish the potential antitumor efficacy of MIP. This study provides evidence into how MIP therapy alters the immunosuppressive tumor milieu to immunologically active one, which ultimately results in tumor regression. There is a need to further understand the MIP mediated modulation of immune response at molecular level. We will further study the use of MIP as an adjunct to chemotherapy since combination therapy is the way to control cancer effectively.
